# Identifying potential therapeutic targets in lung adenocarcinoma: a multi-omics approach integrating bulk and single-cell RNA sequencing with Mendelian randomization

**DOI:** 10.3389/fphar.2024.1433147

**Published:** 2024-07-18

**Authors:** Youpeng Chen, Enzhong Li, Zhenglin Chang, Tingting Zhang, Zhenfeng Song, Haojie Wu, Zhangkai J. Cheng, Baoqing Sun

**Affiliations:** ^1^ Department of Clinical Laboratory, National Center for Respiratory Medicine, National Clinical Research Center for Respiratory Disease, State Key Laboratory of Respiratory Disease, Guangzhou Institute of Respiratory Health, The First Affiliated Hospital of Guangzhou Medical University, Guangzhou Medical University, Guangzhou, China; ^2^ Department of Endocrinology, First Affiliated Hospital of Guangzhou Medical University, Guangzhou, China; ^3^ Department of Obstetrics and Gynecology, The Third Affiliated Hospital of Guangzhou Medical University, Guangzhou, China

**Keywords:** lung adenocarcinoma, bulk RNA sequencing, Mendelian randomization, single-cell RNA sequencing, potential therapeutic targets

## Abstract

Our research aimed to identify new therapeutic targets for Lung adenocarcinoma (LUAD), a major subtype of non-small cell lung cancer known for its low 5-year survival rate of 22%. By employing a comprehensive methodological approach, we analyzed bulk RNA sequencing data from 513 LUAD and 59 non-tumorous tissues, identifying 2,688 differentially expressed genes. Using Mendelian randomization (MR), we identified 74 genes with strong evidence for a causal effect on risk of LUAD. Survival analysis on these genes revealed significant differences in survival rates for 13 of them. Our pathway enrichment analysis highlighted their roles in immune response and cell communication, deepening our understanding. We also utilized single-cell RNA sequencing (scRNA-seq) to uncover cell type-specific gene expression patterns within LUAD, emphasizing the tumor microenvironment’s heterogeneity. Pseudotime analysis further assisted in assessing the heterogeneity of tumor cell populations. Additionally, protein-protein interaction (PPI) network analysis was conducted to evaluate the potential druggability of these identified genes. The culmination of our efforts led to the identification of five genes (tier 1) with the most compelling evidence, including *SECISBP2L*, *PRCD*, *SMAD9*, *C2orf91*, and *HSD17B13*, and eight genes (tier 2) with convincing evidence for their potential as therapeutic targets.

## 1 Introduction

Lung adenocarcinoma (LUAD), the major subtype of non-small cell lung cancer (NSCLC), poses a significant global health challenge (Leiter et al., 2023). With a 5-year survival rate of only 22% (Siegel et al., 2022), there is an urgent need for effective interventions and treatments for LUAD. To address these challenges, it is crucial to identify novel therapeutic targets and biomarkers for LUAD. Genetic studies, particularly those focusing on gene expression, have shown great promise in this regard. Gene expression profiles can provide valuable insights into the molecular mechanisms underlying LUAD pathogenesis and progression, and help identify potential drug targets.

Our research leverages a range of methods, including bulk RNA sequencing, single-cell RNA sequencing (scRNA-seq), and Mendelian randomization (MR). Bulk RNA sequencing has a wide range of uses in cancer classification, biomarker discovery, and optimization of treatments (Dang et al., 2021; Tran et al., 2023). scRNA-seq is critical for understanding tumor transcriptomic programs that are highly heterogeneous (Li and Wang, 2021). MR is a method used to assess the causal relationship between modifiable exposures or risk factors and clinically relevant outcomes (Sekula et al., 2016). Integrating summary data from disease Genome-Wide Association Studies (GWAS) and expression quantitative trait loci (eQTL) studies, MR analysis has been widely employed for repurposing already approved drugs and discovering new therapeutic targets (Gaziano et al., 2021; Storm et al., 2021). The expression level of a gene can be considered a lifelong exposure, and eQTLs located in genomic regions that influence gene expression and are potentially targetable by drugs are often used as proxies for the genes themselves (Zhu et al., 2016; Schmidt et al., 2020).

In this study, we integrated bulk RNA sequencing, scRNA-seq, and MR analyses to systematically identify and prioritize potential therapeutic targets for LUAD. Specifically, we first performed differential gene expression analysis using bulk RNA-seq data to identify genes that are significantly dysregulated in LUAD tissues compared to normal lung tissues. We then applied MR to evaluate the causal effects of the expression levels of these genes on LUAD risk. To further characterize the cellular contexts in which the prioritized genes operate, we conducted scRNA-seq analysis to delineate their cell type-specific expression patterns in the lung microenvironment. Through this integrative approach, we aimed to uncover robust and biologically relevant gene targets that can guide future drug development efforts for LUAD.

In summary, our research employs advanced genomic techniques and causal inference methods to unravel the complex genetic architecture of LUAD, aiming to identify promising drug targets and contribute to the global effort in reducing the burden of this devastating disease. The identified gene targets and their associated biological pathways may serve as a foundation for developing novel therapeutic strategies and precision medicine approaches for LUAD.

## 2 Materials and methods

### 2.1 Study design

**FIGURE 1:**
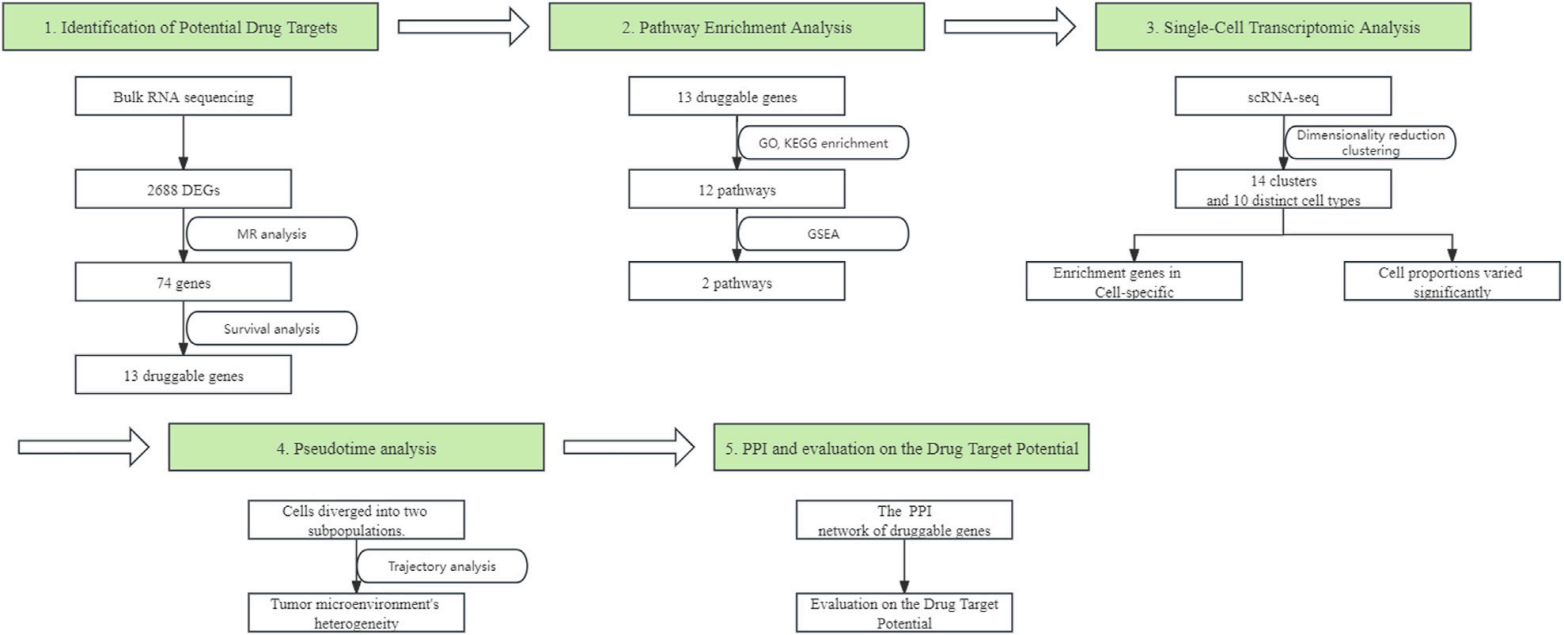
The workflow of designed analysis. DEGs, Differentially expressed genes; MR, Mendelian Randomization; GO, Gene Ontology; KEGG, Kyoto Encyclopedia of Genes and Genomes; GSEA, Gene Set Enrichment Analysis; scRNA-seq, single-cell RNA sequencing; PPI, protein-protein interaction.

In our study, we employed a systematic array of analytical techniques to identify and evaluate potential drug targets for LUAD. We initiated our approach with bulk RNA sequencing to identify differentially expressed genes (DEGs), followed by MR analysis to further narrow down potential therapeutic target genes. Subsequent survival analysis determined the association between the expression levels of these genes and patient survival rates. Pathway enrichment analysis was then conducted to reveal the functional roles of these genes in the disease process. scRNA-seq analysis afforded us insights into the specific expression patterns of these genes across different cell types and the changes in cell proportions within the tumor microenvironment. Pseudotime analysis further delineated the heterogeneity of tumor cell populations across different stages of development. Lastly, by integrating protein-protein interaction (PPI) network analysis, we assessed the potential of these genes as drug targets. The workflow of the study design is presented in Figure 1.

### 2.2 Raw data acquisition

The bulk RNA sequencing data for this study was obtained from the Genomic Data Commons (GDC) TCGA Lung Adenocarcinoma (LUAD) cohort, accessible through the Xena Browser (https://xenabrowser.net/). Alongside this, Phenotype data for a broader set of samples and survival data were also downloaded. These datasets collectively provide a comprehensive genomic profile, encompassing detailed RNA sequencing data, phenotypic characteristics, and survival information from a rigorously quality-controlled and annotated collection of LUAD samples.

In our MR study, we utilized two distinct datasets to explore the genetic underpinnings of LUAD. The first dataset, comprising lung tissue eQTL data, was sourced from European and American participants through the Genotype-Tissue Expression (GTEx) project (https://gtexportal.org/home/datasets/). This dataset was specifically selected to analyze the genetic influences on gene expression in lung tissues of these populations, serving as the exposure variable in our analysis. The second dataset, identified by the ID GCST004744 (McKay et al., 2017) in the European Bioinformatics Institute (EBI) GWAS Catalog (https://www.ebi.ac.uk/gwas/), contains a large collection of genetic variants associated with LUAD risk. This GWAS dataset was employed as the outcome data in our MR analyses, enabling the investigation of potential causal relationships in LUAD. Both datasets were carefully processed and filtered to align with our study’s objectives, ensuring robust and reliable insights into the genetic factors influencing this condition.

10x scRNA-seq data were downloaded from the GSE149655 series. This dataset consists of two LUAD samples and two adjacent non-cancerous tissue samples. The selection of these samples aimed to provide insights into the cellular heterogeneity within LUAD and its surrounding tissue, facilitating a detailed analysis of gene expression patterns at a single-cell level.

### 2.3 Bulk RNA sequencing data processing and analysis

For the analysis of bulk RNA sequencing data, we initially screened our dataset to exclude patients lacking survival data. To identify DEGs, we employed DESeq2 (version 1.40.2) for conducting negative binomial tests in paired comparisons. This analysis was performed using the default settings of the DESeq2 package. we considered genes as differentially expressed if they met the following parameters in the DESeq2 analysis—a Benjamini-Hochberg-adjusted *p*-value of <0.01 and a log2 fold change of >1. We chose a stringent adjusted *p*-value threshold of 0.01 to control for false positives and focus on genes with strong evidence of differential expression, considering the large number of genes being tested simultaneously. This threshold is consistent with recommendations by Schurch et al., 2016, who suggest that a stringent adjusted threshold helps balance false positives and the identification of biologically relevant genes. Additionally, the log2 fold change threshold of >1 was chosen to prioritize genes with a substantial change in expression levels, as suggested by Chen et al., 1000. Additionally, we incorporated an average expression threshold, considering only genes with an average expression greater than 10.

Potential batch effects in our study should also be considered. Batch effects can arise from various sources, such as differences in sample collection, processing, or sequencing protocols, and can introduce systematic biases that confound the biological signals of interest. In our bulk RNA sequencing analysis, we used data from the GDC TCGA-LUAD cohort, which has well-documented procedures for sample collection, processing, and sequencing, minimizing the likelihood of batch effects. Furthermore, we applied the variance stabilizing transformation (vst) function in the DESeq2 package to the DESeqDataSet object to correct for batch effects.

### 2.4 Mendelian randomization (MR) analysis and colocalisation analysis

In our MR study, using the TwoSampleMR package (version 0.5.7), we investigated the causal relationship between gene exposure variables, defined by eQTLs, and LUAD outcomes. Instrumental variables (IVs) were selected from these eQTLs, adhering to a stringent *p*-value threshold of < 5e−08. To ensure the independence of the selected IVs, we performed linkage disequilibrium clumping using the clumping function in the TwoSampleMR package, with an r2 threshold of 0.01. This process identified a set of independent IVs for each gene exposure variable. To assess the relevance of the selected IVs, we calculated the F-statistic for each IV using the formula F = R^2/(1 − R^2) * (n − k − 1)/k, where R^2 = 2 * MAF * (1-MAF) * Beta^2, n is the sample size, k is the number of instrumental variables, and MAF is the minor allele frequency. IVs with an F-statistic greater than 10 were considered relevant and strong instruments for the gene exposure variable, while those with an F-statistic less than 10 were excluded to mitigate weak instrument bias.

To further reduce the potential impact of horizontal pleiotropy on the results of our MR analysis, we selected SNPs within ±1 Mb of the transcription start site of each gene. The harmonise_data function was subsequently applied to harmonize the exposure and outcome datasets, aligning the effect alleles and excluding palindromic SNPs. The present MR study was performed in accord with the recommended items (Supplementary File: STROBE-MR-checklist).

The MR analysis was performed under the following assumptions:

1 Relevance assumption: The genetic variants (IVs) are strongly associated with the exposure (gene expression levels).
2 Independence assumption: The genetic variants (IVs) are not associated with any confounders that influence the relationship between the exposure and the outcome.
3 Exclusion restriction assumption: The genetic variants (IVs) affect the outcome (LUAD risk) only through their effect on the exposure (gene expression levels) and not through any other pathways.

Our MR analysis employed the Wald ratio method for single-SNP analysis and the inverse-variance weighted (IVW) method for analyses involving multiple SNPs. Results were deemed statistically significant at a threshold of *p* < 0.05. We did not apply multiple testing correction to the MR results, as the number of genes being tested was relatively small compared to the bulk RNA-seq analysis. Moreover, the stringent *p*-value threshold used for selecting IVs (P < 5e-08) and the exclusion of SNPs related to LUAD outcomes or associated traits already helped to control for potential false positives.

We conducted a colocalization analysis on SNPs within ±1 Mb of the transcription start site (TSS) for genes potentially causally related to LUAD risk, identified in MR analysis. This analysis was performed using the R package COLOC (version 5.2.2). The parameters were set as P1 = 1 × 10^−4^, P2 = 1 × 10^−4^, and P12 = 1 × 10^−5^. The probability that a given SNP is associated with LUAD risk is denoted as P1; the probability that a given SNP is a significant eQTL is denoted as P2; and the probability that a given SNP is an outcome of both LUAD risk and eQTL is denoted as P12. The COLOC package was used to test five hypotheses, and the posterior probability (PP) was used to quantify support for each hypothesis. The hypotheses were identified as PPH0–PPH4: PPH0, no association with either trait; PPH1, association with the LUAD risk but not the expression of the gene; PPH2, association with the expression of the gene but not the LUAD risk; PPH3, association with the LUAD risk and expression of the gene, with distinct causal variants; and PPH4, association with the LUAD risk and expression of the gene, with a shared causal variant. Due to limited power in the colocalisation analysis, we restricted our analysis to genes reaching a combined PPH3 and PPH4 of ≥0.8 (Giambartolomei et al., 2014).

### 2.5 Evaluation of prognostic value

Genes identified as statistically significant from a MR study were subjected to Kaplan-Meier survival analysis. Patients were classified into high or low expression groups based on the median expression of these genes. Survival differences between these groups were assessed using log-rank tests, with a *p*-value of less than 0.05 considered statistically significant (Dang et al., 2023; Viet-Nhi et al., 2024). We did not apply multiple testing correction to the survival analysis results, as the genes tested were preselected based on their statistical significance in the MR analysis, which already incorporated stringent criteria for selecting instrumental variables (P < 5e-08) and excluded SNPs related to LUAD outcomes or associated traits.

We classified the identified genes into two tiers based on the consistency of their associations with LUAD risk and prognosis across the different analyses. Tier 1 genes were defined as those exhibiting a consistent direction of effect in both the bulk RNA sequencing and MR analyses. Specifically, genes with higher expression in cancer tissues compared to adjacent non-cancerous tissues and a positive association with increased LUAD risk in MR analysis, or genes with lower expression in cancer tissues and a negative association with LUAD risk, were classified as Tier 1. Genes that did not meet these criteria were classified as Tier 2.

### 2.6 Pathway and function enrichment analysis and gene set enrichment analysis

Gene Ontology (GO) and Kyoto Encyclopedia of Genes and Genomes (KEGG) enrichment analyses were conducted on potential therapeutic targets linked to LUAD, utilizing the clusterProfiler (version 4.8.2) and org.Hs.eg.db (version 3.17.0) R packages. For GO enrichment analysis, the background gene set and GO gene sets were obtained from the org.Hs.eg.db package. For KEGG enrichment analysis, the background gene set and KEGG gene sets were obtained from the KEGG database, using the “hsa” (human) organism. Subsequent Gene Set Enrichment Analysis (GSEA) was performed on these significant GO and KEGG pathways. The same gene sets were used for GSEA analysis using the gseGO and gseKEGG functions. For all three analyses, the Benjamini–Hochberg method was applied for multiple testing correction, with an adjusted *p*-value cutoff of 0.05. Pathways achieving an adjusted *p*-value of <0.05 were considered statistically significant.

### 2.7 scRNA-seq data processing and analysis

10 × scRNA-seq data were processed using R, with the Seurat package (version 4.3.0.1) (Macosko et al., 2015) utilized for converting data into Seurat objects. The quality control criteria included a minimum of 3 cells per gene and at least 300 genes per cell, excluding cells with mitochondrial gene content over 10% and hemoglobin gene content over 3%. These thresholds are based on widely accepted practices in the field of scRNA-seq data analysis (Ilicic et al., 2016; Luecken and Theis, 2019) and aim to remove low-quality or potentially damaged cells that may introduce noise into the analysis. Following this, the top 2000 highly variable genes were identified for analysis. Dimensionality reduction and clustering were performed using principal component analysis (PCA). Clustering was then conducted using the Seurat FindClusters function, which applies a shared nearest neighbor modularity optimization-based clustering algorithm. The resolution parameter was set to 0.5, resulting in 14 clusters. These clusters were visualized using Uniform Manifold Approximation and Projection (UMAP) (Becht et al., 2018). Marker gene identification for clusters was conducted with a log2 fold change threshold of 0.25. To assess whether genes are overexpressed in specific cell types, we employed a differential expression analysis based on the Wilcoxon Rank Sum test, comparing gene expression levels between 1 cell type and all other cell types. We define “cell type-specific enrichment” as the significant overexpression of genes in 1 cell type compared to all other cell types. The *p*-values from the Wilcoxon Rank Sum test were adjusted using the Bonferroni correction method to control for multiple testing. Genes with a log2 fold change greater than 0.25 and an adjusted *p*-value less than 0.05 were identified as enriched in a specific cell type. Initial cell type annotation was performed using the “SingleR” package (version 2.2.0) (Aran et al., 2019), with the Human Primary Cell Atlas as a reference. This initial annotation was then refined by examining the expression of known cell type-specific marker genes within each cluster. Clusters expressing similar marker genes were merged, resulting in a final set of 10 distinct cell types. The final annotation was manually confirmed based on marker gene expression, ensuring accurate characterization of cellular subpopulations.

For the scRNA-seq data from the GSE149655 series, we applied batch effect correction using the Seurat package to align the datasets and mitigate potential batch effects. Specifically, we used the “IntegrateData” function, which employs a canonical correlation analysis to identify shared sources of variation between the datasets and then integrates them based on these shared sources. While these measures help to control for batch effects, we cannot entirely rule out their potential influence on our results.

### 2.8 Monocle3 for pseudotime analysis

To investigate the differentiation trajectories within major cell clusters (Type II Alveolar Epithelial Cells, Microvascular Endothelial Cells, and Fibroblasts), we applied the Monocle3 R package (version 1.3.3). For each cell type, the Seurat object was subsetted to include only the cells from the clusters of interest (e.g., clusters 7 and 11 for Fibroblasts) and then converted into a Monocle3 cell_data_set object using the new_cell_data_set function. The data was preprocessed using the preprocess_cds function. Batch effects were corrected using the align_cds function, and then the dimensionality of the data was reduced using the reduce_dimension function. Pseudotime trajectories were inferred using the learn_graph function, and the resulting differentiation pathways were visualized using UMAP plots generated by the plot_cells function. These steps were repeated for each cell type of interest (Type II Alveolar Epithelial Cells, Microvascular Endothelial Cells, and Fibroblasts), with the appropriate cluster IDs used to subset the data and perform pseudotime trajectory analysis.

### 2.9 Protein-protein interaction (PPI) and druggability evaluation

To investigate gene interactions, a PPI network of the potential therapeutic targets was constructed using the STRING database (https://string-db.org/). In the STRING database, we set the minimum required interaction score to medium confidence (0.400) and the species to “*Homo sapiens*.” All other parameters were kept at their default settings. This allowed us to assess the strength and quality of the interactions based on the confidence scores provided by STRING, which are derived from various sources such as experimental data, computational prediction, and text mining. This analysis facilitated the understanding of gene interplay and their biological functions. Simultaneously, we assessed the potential of these genes as therapeutic targets through a literature review. We conducted a systematic search in the PubMed database using the following search strategy: (gene name) AND ((“therapeutic target”) OR (“drug target”) OR (“therapy”) OR (“treatment”)). No restrictions were placed on publication date or article type to ensure comprehensive coverage.

For each gene, we carefully evaluated the evidence in the existing literature to determine its suitability as a therapeutic target for LUAD. We considered the following criteria: 1) the gene’s role in LUAD pathogenesis; 2) the association between gene expression or activity and LUAD prognosis; 3) existing or developing therapeutic approaches targeting the gene or its encoded product; and 4) potential clinical benefits and risks associated with targeting the gene. To further evaluate the therapeutic potential of the identified genes, we cross-referenced our results with three drug-gene interaction databases: DGIdb (Drug Gene Interaction Database) (Freshour et al., 2021), DrugBank (Wishart et al., 2018), and Therapeutic Target Database (Zhou et al., 2024). These databases provide comprehensive information on known and potential drug-target interactions, helping us assess the likelihood of our genes of interest being viable therapeutic targets.

However, our literature review has some limitations. For certain genes, particularly those newly discovered or less studied, there may be insufficient literature evidence to comprehensively assess their therapeutic potential. Moreover, the design quality and reported results of existing studies may vary, posing challenges for cross-study comparison and evidence synthesis. In cases where the literature evidence was inconsistent or sparse, we prioritized the findings from more consistent studies and aimed to present a balanced view of the available evidence. The drug-gene interaction databases also have their own limitations, such as potential biases towards well-studied genes and the inclusion of both validated and predicted interactions. Despite these limitations, our literature review and database cross-referencing provides important preliminary insights into the potential of these genes as therapeutic targets for LUAD and guides future experimental and clinical research directions.

This combined strategy of PPI network construction, drug-gene interaction database analysis and target evaluation provided insights into the therapeutic potential and interaction mechanisms of the potential therapeutic targets.

## 3 Results

### 3.1 Identification of potential drug targets: from differential gene expression to MR and survival analysis

**FIGURE 2:**
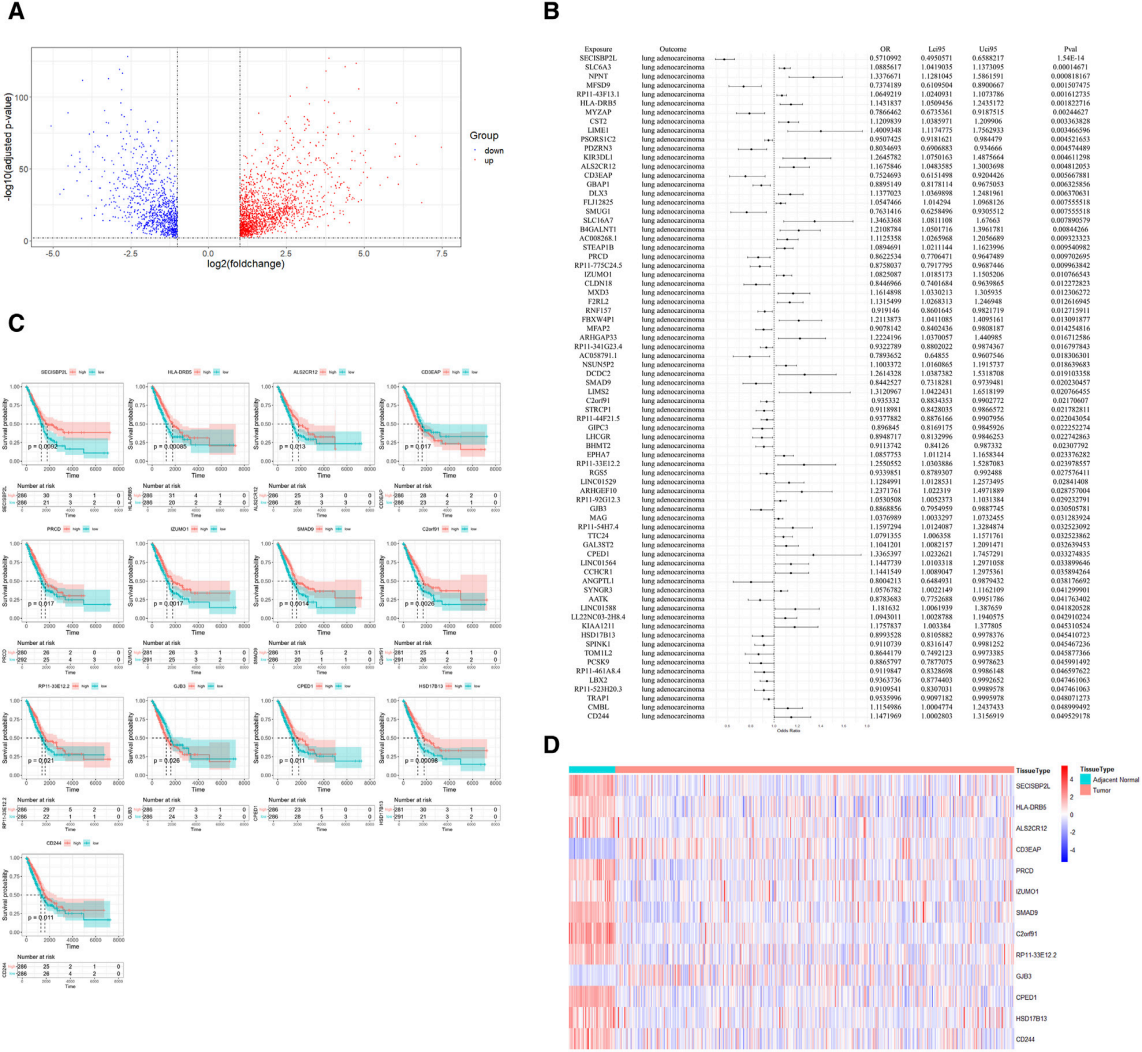
Identification of Potential Drug Targets. **(A)** Volcano plot displaying the differential expression of genes between cancerous and adjacent non-cancerous lung tissues, with upregulated genes highlighted in red, downregulated genes in blue. DEGs were defined as genes with a Benjamini-Hochberg-adjusted *p*-value <0.01 and a log2 fold change >1. **(B)** Forest plot from Mendelian randomization analysis illustrating potential therapeutic targets with lung eQTL data as the exposure and lung adenocarcinoma GWAS data as the outcome. **(C)** Kaplan-Meier survival plots for 13 potential therapeutic targets, where patients with high gene expression (above median) are shown in red and those with low expression (below median) are in blue, with *p*-values denoting statistical significance. **(D)** Heatmap showing the expression profile of potential therapeutic targets in cancerous and adjacent non-cancerous tissues.

In our study, we began by processing bulk RNA sequencing data, excluding patients without survival data. This resulted in a final dataset of 59 adjacent non-tumorous tissues and 513 cancer tissues for our analysis. The differential expression analysis, when intersected with eQTL data, yielded 2,688 DEGs (Supplementary Table S1). Of these, 967 genes were downregulated and 1,721 were upregulated (Figure 2A). This step effectively narrowed down our gene set for more comprehensive analyses.

Subsequently, we utilized MR analysis, with lung eQTL data serving as the exposure and LUAD GWAS data as the outcome. After calculating the F-statistic for each IVs and excluding those with an F-statistic less than 10 (Supplementary Table S2), we identified 74 genes with strong evidence for a causal effect on risk of LUAD. (Figure 2B; Supplementary Table S3). Following the MR analysis, we conducted a colocalization analysis on the SNPs of the 74 genes to further determine the probability that the SNPs associated with LUAD and eQTL share causal genetic variants within the region. The results indicated that most genes and LUAD are likely to share a causal variant within the region (Supplementary Table S4). For further investigation, patients were divided into high and low expression groups based on the median expression levels of these genes.

Through Kaplan-Meier survival analysis, a significant disparity in survival rates was observed in 14 genes, as denoted by a *p*-value less than 0.05. Intriguingly, for 13 of these genes, the variations in survival rates were in alignment with their differential expression between cancerous and adjacent non-cancerous tissues. Genes with higher expression in adjacent non-cancerous tissue compared to cancerous tissue were associated with better survival outcomes. Patients with higher expression of these genes had longer survival times compared to those with lower expression (Figures 2C,D). This finding underscores the prognostic significance of these genes in LUAD.

Based on the consistency of their associations across the different analyses, we classified the 13 identified genes into two tiers. Five genes (*SECISBP2L*, *PRCD*, *SMAD9*, *C2orf91*, and *HSD17B13*) were classified as Tier 1, exhibiting consistent directions of effect in both the bulk RNA sequencing and MR analyses. The remaining eight genes were classified as Tier 2.

### 3.2 Comprehensive pathway enrichment analysis of the 13 prognostic differential genes: GO, KEGG, and GSEA approaches

**FIGURE 3:**
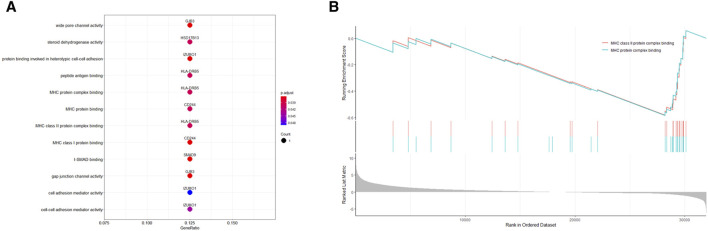
Pathway enrichment analysis of potential therapeutic targets in lung adenocarcinoma. **(A)** Gene Ontology (GO) enrichment analysis showing significant molecular function pathways among the 13 potential therapeutic targets. Key pathways are related to immune response, including MHC class I and II protein bindings, gap junction channel activity, and cell-cell adhesion. **(B)** Gene Set Enrichment Analysis (GSEA) results indicating the significance of the “MHC Class II Protein Complex Binding” and “MHC Protein Complex Binding” pathways across the entire dataset.

In our comprehensive pathway enrichment analysis, we sought to elucidate the functional impact of the 13 potential therapeutic targets identified in LUAD. GO enrichment analysis using all human genes from the org.Hs.eg.db package as the background was performed (Supplementary Tables S5–7), which revealed no statistically significant pathways within the Biological Process (BP) and Cellular Component (CC) categories. However, within the Molecular Function (MF) category, 12 pathways were found to be statistically significant (Figure 3A). These pathways were predominantly related to immune response mechanisms, such as MHC class I and II protein bindings, essential for antigen presentation. Additionally, pathways involved in cellular communication and adhesion, including gap junction channel activity and cell-cell adhesion, were significantly enriched, underscoring their importance in the regulation of cell proliferation and the maintenance of tissue architecture. Furthermore, enrichment in steroid dehydrogenase activity and TGF-β signaling, as evidenced by I-SMAD binding, indicates a notable involvement in hormone metabolism pathways.

Contrastingly, KEGG enrichment analysis using the human-specific KEGG gene sets did not yield any pathways of statistical significance (Supplementary Table S8). To gain a more robust perspective of the dataset, GSEA was subsequently employed using the same gene sets from GO and KEGG (Supplementary Tables S9–12). GSEA demonstrated that out of the 12 meaningful pathways identified by GO analysis, only two consistently exhibited gene expression changes across the entire dataset when considering overall expression data, not limited to potential therapeutic targets. This finding highlights the potential relevance of these particular pathways, enhancing our understanding of their role in LUAD. Specifically, the pathways “MHC Class II Protein Complex Binding” and “MHC Protein Complex Binding” were validated by GSEA, suggesting their critical involvement in the disease’s molecular landscape (Figure 3B).

### 3.3 Single-cell transcriptomic analysis: unveiling cell type-specific expression of prognostic differential genes

**FIGURE 4:**
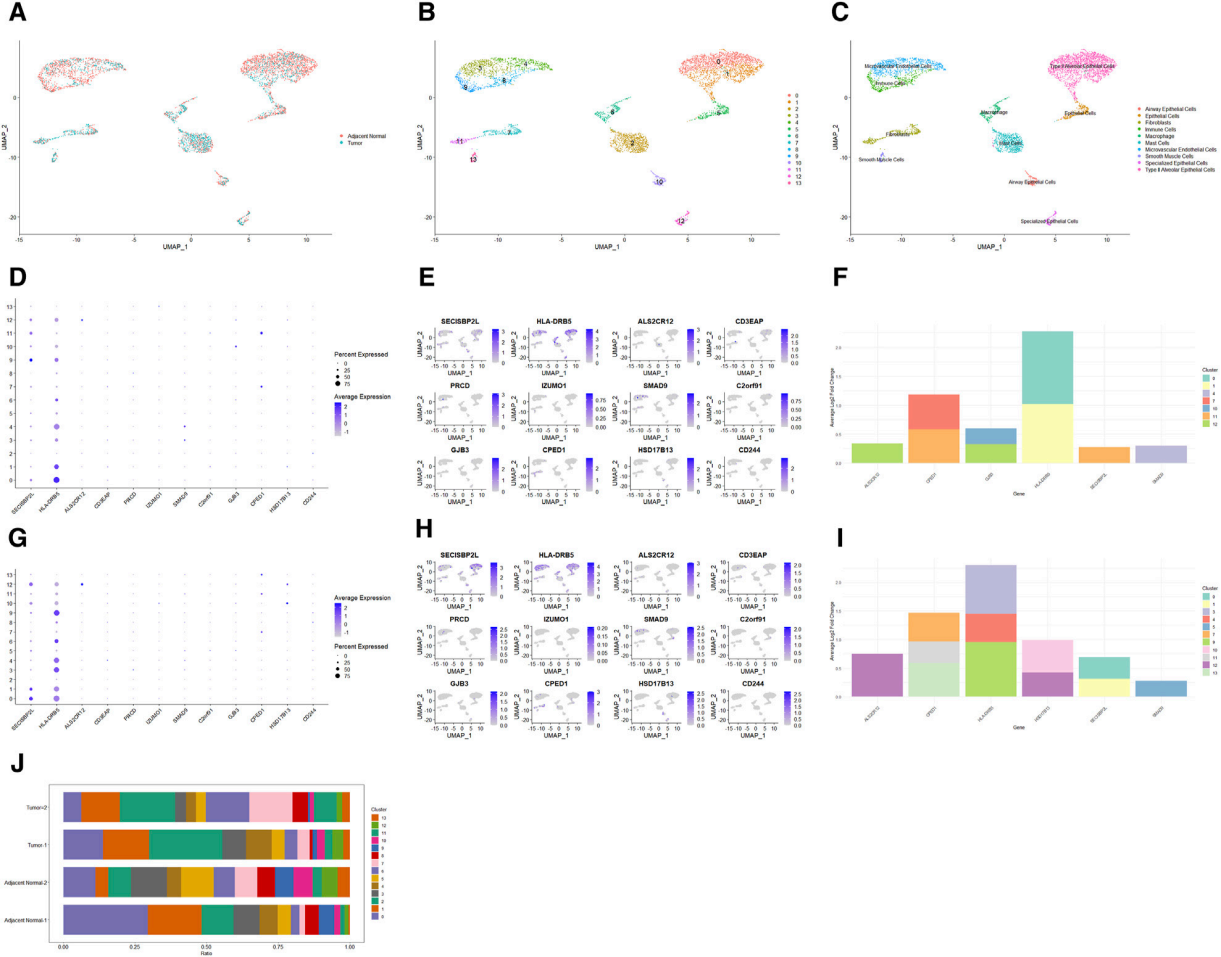
Single-Cell Transcriptomic Analysis of Potential Therapeutic Targets in Lung Adenocarcinoma and Adjacent Normal Tissues. **(A, B)** UMAP plots depicting 14 distinct cell clusters identified within lung adenocarcinoma and adjacent normal tissues. **(C)** UMAP plot highlighting the distribution of 10 cell types across the identified clusters. **(D, E)** Bubble and UMAP plots showing the expression and distribution of 12 out of 13 potential therapeutic targets in cancer tissues, with the absence of detection for RP11-33E12.2. **(F)** Stacked bar graph indicating the cell type-specific enrichment of 6 potential therapeutic targets in lung adenocarcinoma. **(G, H)** Bubble and UMAP plots depicting the expression and distribution of 12 out of 13 potential therapeutic targets in adjacent normal tissues, with RP11-33E12.2 again not detected. **(I)** Stacked bar graph demonstrating the cell type-specific enrichment of 6 potential therapeutic targets in normal lung tissues, contrasting with the expression patterns observed in cancerous tissues. **(J)** Stacked bar chart comparing the cellular proportions of two cancer tissues and two adjacent normal tissues across the identified clusters, emphasizing the differential cellular composition between the two conditions.

To explore the cell type-specific enrichment of 13 potential therapeutic targets, we performed a scRNA-seq analysis. This resulted in the identification of 14 distinct clusters (Figures 4A,B), which were visualized using UMAP. The clusters were further annotated by SingleR and manual refinement based on marker gene expression, identifying 10 main cell types: Type II Alveolar Epithelial Cells, Mast Cells, Microvascular Endothelial Cells, Epithelial Cells, Macrophage, Fibroblasts, Immune Cells, Airway Epithelial Cells, Specialized Epithelial Cells, and Smooth Muscle Cells (Figure 4C). Of the 13 genes, 12 were expressed in LUAD tissues, with *RP11-33E12.2* not detected (Figures 4D,E). Notably, 6 genes exhibited cell type-specific enrichment in LUAD tissue (Figure 4F). *ALS2CR12* was predominantly enriched in Specialized Epithelial Cells. *CPED1* showed significant enrichment in Fibroblasts. *HLA-DRB5* was mainly enriched in Type II Alveolar Epithelial Cells, while *SECISBP2L* was found in Fibroblasts (Cluster 11). *SMAD9* showed enrichment in Microvascular Endothelial Cells (Cluster 4). *GJB3*, unique to cancer tissue compared to adjacent normal tissue, was enriched in both Specialized Epithelial Cells and Airway Epithelial Cells.

In adjacent normal lung tissue, 12 out of the 13 genes were expressed, with *RP11-33E12.2* again undetected (Figures 4G,H). Six genes showed cell type-specific enrichment in adjacent normal lung tissues (Figure 4I). *ALS2CR12* exhibited a notable enrichment in Specialized Epithelial Cells, with its abundance being significantly higher compared to cancer tissues, indicated by a greater log2 fold change. *CPED1* was enriched in both Fibroblasts and Smooth Muscle Cells. Interestingly, *HLA-DRB5* was enriched in Microvascular Endothelial Cells and Immune Cells (Cluster 9), and *SECISBP2L* in Type II Alveolar Epithelial Cells. *SMAD9* was enriched in Epithelial Cells. *HSD17B13*, unique to adjacent normal tissue compared to cancer tissue, was enriched in both Specialized Epithelial Cells and Airway Epithelial Cells.

Additionally, the cellular proportions in two cancer tissues and two adjacent normal tissues differed significantly (Figure 4J). In adjacent normal tissues, clusters 0 (Type II Alveolar Epithelial Cells) and 3 (Microvascular Endothelial Cells) had a higher proportion compared to clusters 1 (Type II Alveolar Epithelial Cells) and 4 (Microvascular Endothelial Cells), respectively, while in cancer tissues, the proportions were opposite or similar.

### 3.4 Characterizing differentiation dynamics in major cell populations through pseudo-temporal sequencing

**FIGURE 5:**
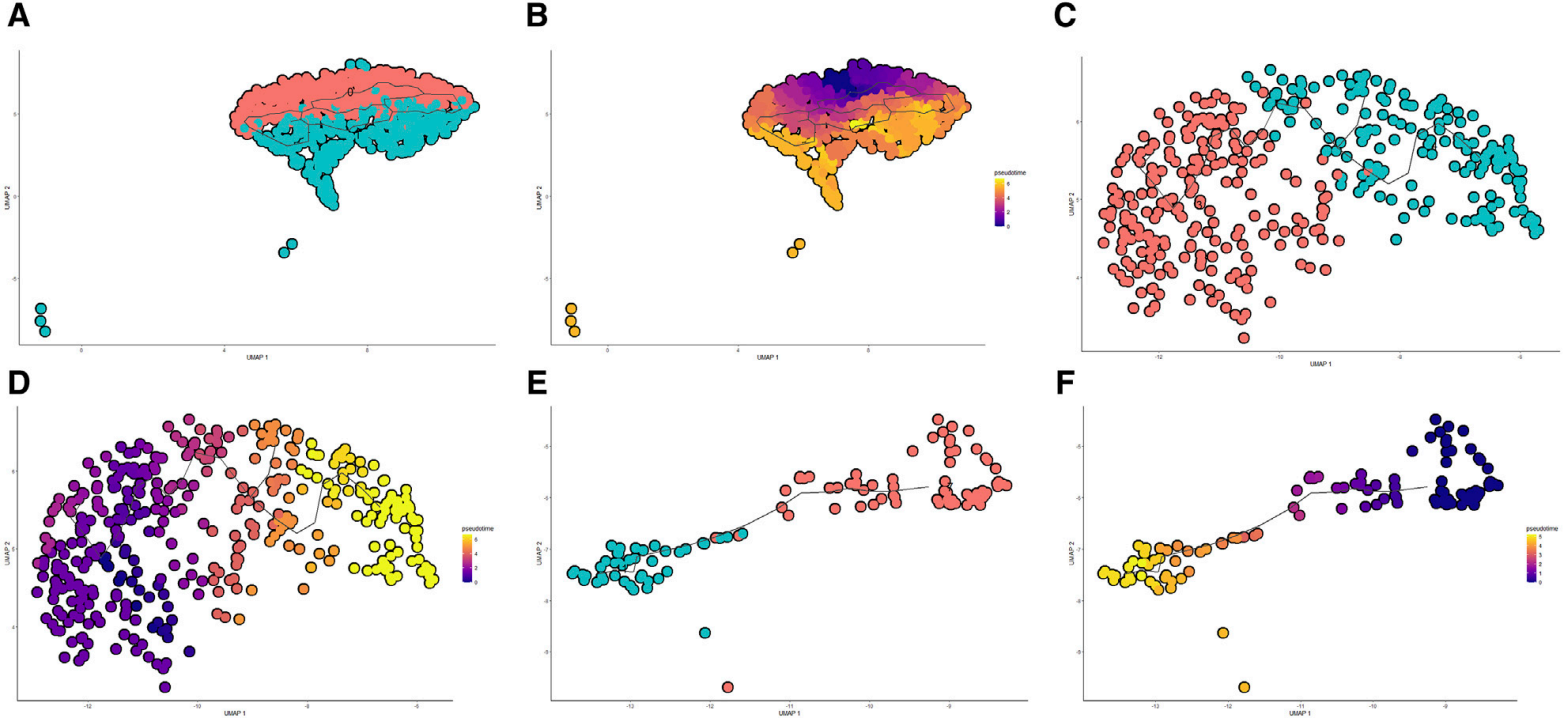
Pseudo-temporal Analysis of Cell Differentiation in Lung Adenocarcinoma. **(A, B)** UMAP visualization and pseudo-temporal trajectory of Type II Alveolar Epithelial Cells showing the progression from cluster 0 to cluster 1, depicting the potential early to late differentiation stages within the tumor microenvironment. **(C, D)** UMAP and trajectory analysis of Microvascular Endothelial Cells transitioning from cluster 3 to cluster 4, reflecting changes in the cellular landscape associated with tumor evolution. **(E, F)** UMAP plots coupled with pseudo-temporal ordering of Fibroblasts, charting a course from cluster 7 to cluster 11, illustrating a progression of fibroblast differentiation within lung adenocarcinoma.

Through pseudo-temporal sequencing analysis, we have discerned the heterogeneity of cell states in LUAD progression, where specific genes exhibited significant correlations with the transitions in distinct cell types, suggesting their potential as therapeutic targets. Dimensionality reduction and clustering of scRNA-seq data revealed bifurcation in three major cell populations—Type II Alveolar Epithelial Cells, Microvascular Endothelial Cells, and Fibroblasts—each diverging into two subgroups, indicative of the tumor microenvironment’s heterogeneity. Monocle 3 trajectory analysis charted the developmental courses within these cell types: Type II Alveolar Epithelial Cells transitioned from cluster 0 to cluster 1 (Figures 5A,B), Microvascular Endothelial Cells from cluster 3 to cluster 4 (Figures 5C,D), and Fibroblasts from cluster 7 to cluster 11 (Figures 5E,F). These differentiation pathways echo the cellular composition differences between cancerous and adjacent normal tissues, particularly an increased proportion of cells in clusters 1 and 4 within the tumorous tissues, pointing to a dynamic interplay between cell differentiation and tumor evolution.

Intriguingly, the trajectory analysis revealed differential expression patterns of key potential therapeutic target genes along these developmental trajectories. *HLA-DRB5*, a gene previously identified as downregulated in cancer tissues compared to adjacent normal tissues in our bulk RNA sequencing analysis, exhibited a decrease in expression as Type II Alveolar Epithelial Cells transitioned from cluster 0 to cluster 1 and as Microvascular Endothelial Cells progressed from cluster 3 to cluster 4. Similarly, *CPED1*, another gene with lower expression in cancer tissues, showed reduced expression during the progression of Fibroblasts from cluster 7 to cluster 11. These findings provide a more detailed view of the cell type-specific expression changes of these potential therapeutic targets, further supporting their involvement in LUAD progression and their potential as intervention points.

The integration of our pseudo-temporal analysis with the previous bulk RNA sequencing results and cell type-specific enrichment findings paints a more comprehensive picture of the complex gene expression landscape in LUAD. The observed downregulation of *HLA-DRB5* in both Type II Alveolar Epithelial Cells and Microvascular Endothelial Cells during their developmental trajectories, along with the reduced expression of *CPED1* in Fibroblasts, is consistent with their overall reduced expression in cancer tissues, highlighting their potential significance in LUAD pathogenesis. Further exploration of these genes and their associated pathways within the context of cellular differentiation may yield valuable insights into the mechanisms driving LUAD progression and guide the development of targeted therapeutic strategies.

### 3.5 PPI and evaluation on the drug target potential

In the present study, we analyzed the PPI network of potential therapeutic targets. Our investigation revealed limited interactions among these genes, with interactions observed only between *HLA-DRB5* and *CD244*, and *HSD17B13* and *CD244*. Notably, the expression levels of these genes of tumorous tissues were found to be downregulated in comparison to adjacent non-tumorous tissues in LUAD (Supplementary Figure S1). *HLA-DRB5* and *CD244* play pivotal roles in controlling antigen processing and presentation, as well as T-cell activation. It has been observed that a downregulation in the expression of *HLA-DRB5* correlates with a poor prognosis in LUAD (Sun et al., 2021; Wu et al., 2021; Wang et al., 2022; Xu et al., 2022). Additionally, in NSCLC patients, *CD244* has been identified as a potential negative prognostic biomarker (Vaes et al., 2021).

In our assessment of the druggability of the 13 potential therapeutic target genes, we found that four of these genes (*HLA-DRB5*, *GJB3*, *HSD17B13*, and *CD244*) have been targeted for drug development (Supplementary Table S13). For *HLA-DRB5*, several drugs have been identified, including 1D09C3 for various forms of cancer, Coccidioides immitis spherule for detecting delayed-type hypersensitivity in individuals with a history of pulmonary coccidioidomycosis, and Apolizumab for solid tumors/cancer. *GJB3* has been associated with drugs such as SRT1720, which has potential applications in treating conditions related to aging and metabolic diseases, and SELISISTAT, which is being explored for the treatment of Huntington’s disease.

A drug targeting *HSD17B13*, INI-822, has been found to be applicable for fibrotic liver diseases, including non-alcoholic steatohepatitis, and is currently undergoing Phase I clinical trials. Moreover, Compound BI-3231 represents a novel, effective, and selective inhibitor of HSD17B13 (Thamm et al., 2023). ARO-HSD is specifically designed to target hepatocytes, aiming to silence HSD17B13 expression (Mak et al., 2023). Additionally, GSK4532990 has been identified as a potential drug targeting *HSD17B13* for the treatment of non-alcoholic steatohepatitis.

Furthermore, treatment with an anti-CD244 monoclonal antibody significantly impaired the growth of established HNSCC tumors in wild-type mice and increased the infiltration of CD8^+^ T cells within the tumors (Agresta et al., 2020). A vaccine targeting CD244, leveraging antibodies against CD244 or its natural ligand CD48, has been developed to enhance T-cell activation, offering a novel approach against infectious diseases and cancers. Elotuzumab, indicated in combination with lenalidomide and dexamethasone for the treatment of patients with multiple myeloma who have received one to three prior therapies, and ALDESLEUKIN, an antineoplastic agent, have also been identified as drugs targeting CD244.

## 4 Discussion

The development of therapeutics for LUAD presents significant challenges, primarily due to the complexity of Phase I trials and resistance to traditional therapies. Conventional treatments like radiation, surgery, and chemotherapy often lead to adverse side effects (Lin, 2019; Rohilla et al., 2023). Employing our multi-tiered approach, we identified 13 potentially potential therapeutic targets that may influence the outcome of LUAD. Notably, among these 13 potential therapeutic targets, we discovered five with the most compelling evidence (tier 1): *SECISBP2L*, *PRCD*, *SMAD9*, *C2orf91*, and *HSD17B13*. These genes demonstrated a consistent causal effect on LUAD risk in MR analysis, and were also found to be differentially expressed between cancerous and adjacent non-cancerous tissues in bulk RNA sequencing. Specifically, genes showing a positive causal effect on LUAD risk in MR analysis were upregulated in cancer tissues compared to adjacent tissues, while genes with a negative causal effect were downregulated. Additionally, we identified eight genes with convincing evidence for druggability (tier 2).

The classification of genes into tiers based on the consistency of their associations across different analyses provides a prioritized list of potential therapeutic targets for LUAD. The Tier 1 genes, with their robust and consistent associations, represent the most promising candidates for further validation and drug development efforts. The Tier 2 genes, while still exhibiting convincing evidence for druggability, may require additional investigation to elucidate their roles in LUAD pathogenesis and assess their suitability as therapeutic targets. Inconsistencies between Tier 1 and Tier 2 genes may arise from the complexity of LUAD pathogenesis and limitations of the analytical methods. For instance, bulk RNA sequencing provides an average gene expression profile across all cells in a tissue sample, which may obscure cell type-specific expression patterns. In contrast, MR analysis assesses the causal relationship between gene expression and disease risk based on genetic variations, which may not fully capture the dynamic nature of gene expression regulation. Moreover, survival analysis focuses on the association between gene expression and patient outcomes, which can be influenced by various clinical factors beyond the gene itself. These methodological differences may contribute to the observed inconsistencies between the tiers. Despite these challenges, our multi-tiered approach helps prioritize the most promising therapeutic targets. Future studies should aim to validate these findings in larger, independent cohorts and employ additional experimental approaches to elucidate the biological mechanisms underlying the gene-disease associations.

Previous studies have shown downregulation of *SECISBP2L* and *HLA-DRB5* in lung cancer (McKay et al., 2017), with *HLA-DRB5* expression inversely correlated with LUAD risk (Xu et al., 2022). *ALS2CR12* and *GJB3* have been associated with lung cancer (Fehringer et al., 2016; Cheng et al., 2023), with *GJB3* predictive of survival rates in LUAD patients. *CD3EAP*, *SMAD9*, and *CD244* are considered valuable tumor markers (Yin et al., 2013; Hou et al., 2017; Yu et al., 2018; Pan et al., 2020; Yin et al., 2020; Gao et al., 2021; Vaes et al., 2021; Yin et al., 2022), with *CD3EAP* particularly noteworthy as a tumor marker in smokers. *CPED1* is regarded as one of the tumor suppressor genes in LUAD (Hsu et al., 2017). We also discovered several novel candidate potential therapeutic targets, including *PRCD*, *IZUMO1*, *C2orf91*, *RP11-33E12.2*, and *HSD17B13*. Among these, *PRCD*, *C2orf91*, and *HSD17B13* (tier 1) were prioritized as the most compelling due to the robust evidence supporting their potential impact on LUAD, thus warranting further exploration. *PRCD* is a key gene in the development of retinal photoreceptor cells, crucial for the high fidelity of photoreceptor disc formation (Spencer et al., 2019). Retinas lacking *PRCD* produce an abundance of extracellular vesicles containing rhodopsin, and discs fail to form properly (Allon et al., 2019; Sechrest et al., 2020). *C2orf91*, located on human chromosome 2, has been implicated in ALK-positive NSCLC, showing sensitivity to alectinib in ALK-C2orf91(intergenic) (Yan et al., 2023). *HSD17B13* belongs to the HSD17B family, exhibiting NAD(P)H/NAD(P)+ dependent oxidoreductase activity. Current research primarily focuses on non-alcoholic fatty liver disease (Ferenci et al., 2019; Ma et al., 2019; Stickel et al., 2020; Ioannou, 2021; Zhang et al., 2021; Luukkonen et al., 2023).

In our pathway enrichment analysis, we found that these genes are predominantly enriched in pathways related to immune response mechanisms. Considering the overall expression data, pathways such as “MHC class II protein complex binding” and “MHC protein complex binding” were validated through GSEA, indicating their critical roles in the molecular landscape of the disease, potentially linked to increased immune evasion of the tumor. Additionally, pathways involved in cell communication and adhesion were also highlighted, emphasizing their importance in regulating cell proliferation and maintaining tissue architecture. To further understand the specific expression patterns of these genes in different cell types and the heterogeneity within the tumor microenvironment, we conducted scRNA-seq analysis. Our findings reveal that six of the 13 genes (*ALS2CR12*, *CPED1*, *HLA-DRB5*, *SECISBP2L*, *SMAD9*, *GJB3*) show cell type-specific enrichment in cancerous tissues, with GJB3 being unique to cancer tissues. Furthermore, six genes (*ALS2CR12*, *CPED1*, *HLA-DRB5*, *SECISBP2L*, *SMAD9*, *HSD17B13*) demonstrate cell type-specific enrichment in adjacent non-cancerous tissue, with *HSD17B13* uniquely expressed in these tissues. Interestingly, one of the 13 genes, *RP11-33E12.2*, was not detected in either cancerous or adjacent non-cancerous tissues in our scRNA-seq analysis. This could be due to several reasons. First, *RP11-33E12.2* might be expressed at very low levels, below the detection threshold of the scRNA-seq technology used. Second, *RP11-33E12.2* might be expressed in a rare cell type that was not well represented in our scRNA-seq data. Third, there could be technical issues with the scRNA-seq data that prevented the detection of this gene. Despite the absence of *RP11-33E12.2* in the scRNA-seq data, its identification as a potential therapeutic target in our bulk RNA sequencing, MR, and survival analyses suggests that it may still play a role in LUAD pathogenesis. Further studies using more sensitive techniques, such as qPCR or RNA *in situ* hybridization, could help validate the expression and function of *RP11-33E12.2* in LUAD tissues. In adjacent normal lung tissue, *ALS2CR12* exhibited a notable enrichment in Specialized Epithelial Cells, with its abundance being significantly higher compared to cancer tissues, indicated by a greater log2 fold change. Interestingly, Type II Alveolar Epithelial Cells specifically enrich for the *SECISBP2L* gene in adjacent non-cancerous tissues, while *HLA-DRB5* gene is enriched in cancer tissues. Differences in cell proportions between tissues and trajectory analyses showed that for genes downregulated in cancerous tissues (as identified in bulk RNA sequencing), certain cell types (Type II Alveolar Epithelial Cells, Microvascular Endothelial Cells, and Fibroblasts) tend to form subgroups with even lower gene expression, and the proportion of these subgroups is increased in cancerous tissues. Therefore, these findings support the therapeutic targets identified in our study.

The STRING database is a robust resource for protein-protein interactions, but it has limitations such as potential biases towards well-studied proteins and interactions. Its integration of data from various sources can lead to an overrepresentation of certain interactions and varying quality of reported interactions, which should be considered when interpreting our PPI network analysis results. Our PPI network analysis identified key interactions between *HLA-DRB5* and *CD244*, as well as *HSD17B13* and *CD244*, as potential therapeutic targets in LUAD, with these genes showing downregulation in tumor tissues. The *HLA-DRB5*-*CD244* interaction is particularly noteworthy due to their roles in immune response regulation, suggesting a possible contribution to immune evasion in LUAD. Similarly, the *HSD17B13*-*CD244* interaction introduces a novel aspect to LUAD pathogenesis, given *HSD17B13*’s known association with liver diseases and its potential druggability in lung cancer. The identification of specific drugs targeting *HLA-DRB5*, *GJB3*, *HSD17B13*, and *CD244* for various indications, such as cancer, metabolic diseases, and liver disorders, further strengthens their potential as therapeutic targets in LUAD. These findings underscore the importance of further research to explore the functional impacts of these interactions and their therapeutic potential in LUAD, including the possibility of combination therapies targeting these gene interactions. The identification of specific drugs targeting these genes provides valuable leads for further preclinical and clinical studies to evaluate their efficacy and safety in the context of LUAD treatment.

In our investigation of potential therapeutic targets for LUAD, we employed a multifaceted approach, including bulk RNA sequencing, MR, survival analysis, pathway enrichment, scRNA-seq, pseudotime analysis, and PPI network analysis. The consistency of results across multiple rigorous analyses affirmed the robustness of our findings. Additional evidence from single-cell type expression analysis, PPI, and druggability assessments provided insights into the potential pathogenic roles of potential therapeutic targets in LUAD and further prioritized potential therapeutic targets. However, our study has several limitations. First, our sample size for scRNA-seq was relatively small, which may limit the generalizability of our findings. Future studies with larger cohorts are needed to validate our results. Second, while we employed multiple analytical methods to identify and prioritize potential therapeutic targets, experimental validation of these targets was not performed in this study. *In vitro* and *in vivo* functional studies are necessary to confirm the roles of these genes in LUAD pathogenesis and their potential as therapeutic targets. Third, our study focused on gene expression data and genetic associations. Integration of other omics data, such as proteomics and metabolomics, could provide a more comprehensive understanding of the molecular mechanisms underlying LUAD. Fourth, our pathway enrichment and PPI network analyses were based on current knowledge databases, which may not capture all relevant biological interactions. Additionally, these databases may have inherent biases, such as an overrepresentation of well-studied pathways and interactions, which could influence the results of our analyses. As these databases continue to expand, reanalysis of our data may provide additional insights. Potential sources of bias in our MR analysis, such as population stratification and pleiotropy, should also be considered. To mitigate population stratification, we used summary statistics from the GTEx project and the GWAS Catalog (GCST0047449), both of which include samples from individuals of European ancestry. To address pleiotropy, we selected SNPs within ±1 Mb of the transcription start site of each gene and performed a colocalization analysis. However, residual pleiotropy may still affect our results. Due to the limited number of SNPs available for each gene, we were unable to employ more advanced MR methods, such as MR-Egger regression, to further account for potential pleiotropic effects. Finally, it is important to acknowledge the potential limitations of the DESeq2 method used for differential gene expression analysis. DESeq2 assumes that the majority of genes are not differentially expressed between conditions and that the count data follows a negative binomial distribution. Violation of these assumptions may impact the accuracy of the results. Additionally, DESeq2 relies on a generalized linear model to estimate the coefficients for each gene, which may be sensitive to outliers or extreme values. To mitigate these limitations, we applied stringent quality control measures to ensure compliance with the assumptions of DESeq2. Furthermore, potential biases inherent in the datasets used in our study, such as GDC, GTEx, EBI GWAS Catalog, and GSE149655 series, should be considered. For instance, the GTEx data is primarily derived from European and American participants, which may not fully represent the global population. Additionally, the relatively small sample size in the GSE149655 series may limit the generalizability of our findings. Despite these potential biases, we have taken measures to ensure the robustness of our analyses, such as applying stringent quality control procedures, employing strict thresholds for selecting instrumental variables, and conducting a comprehensive literature review and drug-gene interaction database cross-referencing to validate our findings.

In conclusion, our study identified several promising therapeutic targets for LUAD through a multi-tiered approach integrating bulk RNA sequencing, MR, survival analysis, pathway enrichment, scRNA-seq, pseudotime analysis, and PPI network analysis. While our findings provide valuable insights into the molecular mechanisms of LUAD and potential therapeutic strategies, further experimental and clinical research is needed to evaluate the practicability and efficacy of these candidate targets, in order to confirm our current findings and advance their translational applications.

## Data Availability

The data in the study are deposited in the following repositories: 1) Bulk RNA sequencing data, phenotype data, and survival data: Genomic Data Commons (GDC) TCGA Lung Adenocarcinoma (LUAD) cohort, accessible through the Xena Browser (https://xenabrowser.net/), Dataset name: GDC TCGA Lung Adenocarcinoma (LUAD). Data types: Gene expression RNAseq, phenotype data, and survival data. 2) Lung tissue eQTL data: Genotype-Tissue Expression (GTEx) project (https://gtexportal.org/home/datasets/). Dataset name: GTEx Analysis V8. Data type: Lung tissue eQTL data. 3) GWAS data for LUAD risk: European Bioinformatics Institute (EBI) GWAS Catalog, accession number GCST004744 (https://www.ebi.ac.uk/gwas/). 4) 10x scRNA-seq data: Gene Expression Omnibus (GEO), accession number GSE149655.

## Abbreviations

BP, Biological Process; CC, Cellular Component; DEGs, Differentially expressed genes; EBI, European Bioinformatics Institute; GDC, Genomic Data Commons; GO, Gene Ontology; GSEA, Gene Set Enrichment Analysis; GTEx, Genotype-Tissue Expression; GWAS, Genome-Wide Association Studies; IVW, Inverse-variance weighted; IVs, Instrumental variables; KEGG, Kyoto Encyclopedia of Genes and Genomes; LUAD, Lung adenocarcinoma; MF, Molecular Function; MR, Mendelian randomization; NSCLC, Non-small cell lung cancer; PCA, Principal component analysis; PPI, protein-protein interaction; UMAP, Uniform Manifold Approximation and Projection; eQTL, expression quantitative trait loci; scRNA-seq, single-cell RNA sequencing.
