# Supplementary figures and images for: Identifying potential therapeutic targets in lung adenocarcinoma: a multi-omics approach integrating bulk and single-cell RNA sequencing with Mendelian randomization
Source: Front Pharmacol. 2024 Jul 18;15:1433147. doi: 10.3389/fphar.2024.1433147 (PMC11291359; doi:10.3389/fphar.2024.1433147)

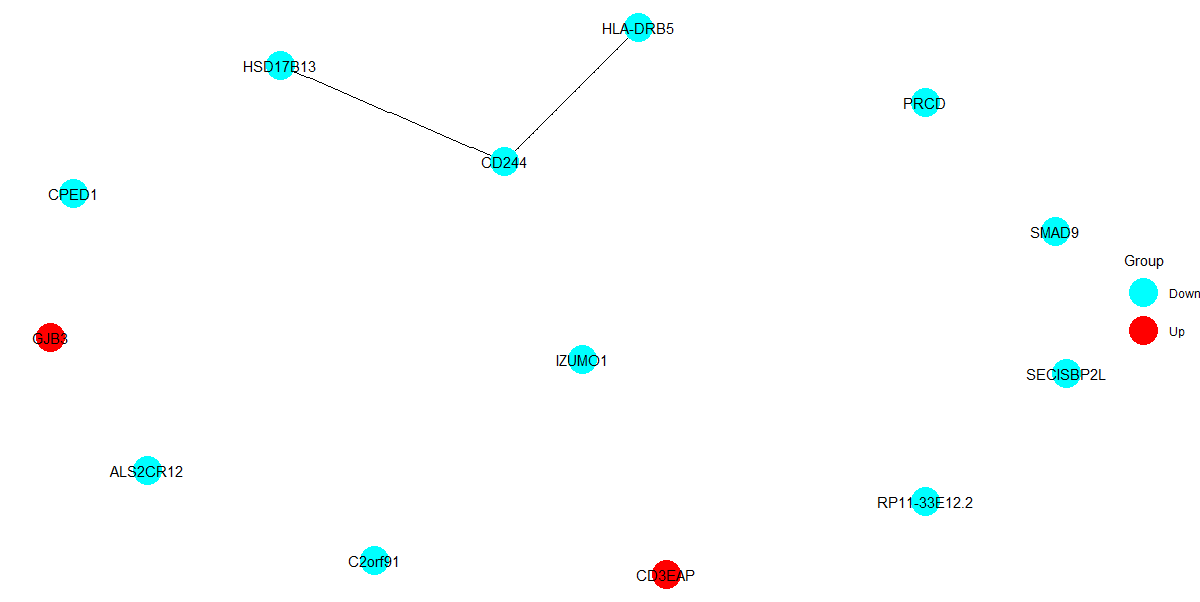

Supplement: Supplementary file 9 [file Image1.PNG]
